# Birth weight charts for a Chinese population: an observational study of routine newborn weight data from Chongqing

**DOI:** 10.1186/s12887-019-1816-9

**Published:** 2019-11-11

**Authors:** Xue Zhao, Yinyin Xia, Hua Zhang, Philip N. Baker, Tom Norris

**Affiliations:** 1 Department of Obstetrics and Gynaecology, The First Affiliated Hospital of Chongqing Medical University, No. 1 Youyi Road, Yuzhong District, Chongqing, 400016 People’s Republic of China; 2 School of Public Health and Management, Chongqing Medical University, Chongqing, China; 3 Canada–China–New Zealand Joint Laboratory of Maternal and Fetal Medicine, Chongqing Medical University, Chongqing, China; 4 College of Life Sciences, University of Leicester, Leicester, UK; 5 School of Sport, Exercise and Health Sciences, National Centre for Sport and Exercise Medicine, Loughborough University, Loughborough, UK

**Keywords:** Birth weight centiles, China, LMS, Urban, Rural

## Abstract

**Background:**

To construct birth weight charts for the Chongqing municipality, China and to identify whether differences in birth weight exist across urban/rural populations, thereby warranting separate charts.

**Methods:**

Secondary analysis of routinely collected data from 338,454 live infants between 2014 and 2017 in Chongqing municipality. Sex-specific birth weight-for-gestational age centiles were constructed by the lambda-mu-sigma method via the GAMLSS R-based package. This method remodels the skewed birth weight distribution to estimate a normal distribution, allowing any birth weight centile to be generated. A separate set of centiles were created, accounting for urban/rural differences in birth weight.

**Results:**

The centiles performed well across all gestational ages. For example, 2.37% (*n* = 4176) of males and 2.26% (*n* = 3656) of females were classified as below the 2nd centile (expected percentage = 2.28%), 49.75% of males (*n* = 87,756) and 50.73% of females (*n* = 82,203) were classified as below the 50th centile (expected proportion = 50%) and 97.52% of males (*n* = 172,021) and 97.48% of females (*n* = 157,967) were classified as below the 98th centile (expected proportion = 97.72%). The overall estimated centiles of birth weight for rural infants were higher than the centiles for urban infants at the earlier gestational ages (< 37 gestational weeks). However, this trend was reversed in infants born at term.

**Conclusion:**

We have constructed a readily utilizable set of birth weight references from a large representative sample of births in Chongqing. The method used to construct the references allows for the calculation of the exact centile for any infant delivered between 28 and 42 completed weeks, which was not possible with previous charts.

## Background

Birth weight is a key measure of infant health, providing an indication of prenatal wellbeing, risk of postnatal complications and survival [1]. The identification of infants who may have experienced intrauterine growth restriction (IUGR) and are thus at an increased risk of adverse neonatal outcomes is a central component of neonatal surveillance. An informative parameter is birth weight adjusted for gestational age. Centile reference charts of smoothed birth weight curves across gestational period are routinely used in clinical practice. Infants with birth weights which fall in the extremes of the centile distribution (e.g. <10th centile or > 90th centile) are subject to increased monitoring in the hope of identifying those who may be at risk of complications. Infants whose birth weight for gestational age falls below the 10th centile of the reference population are classified as small-for-gestational age (SGA), with this phenotype conferring an increased risk of various adverse neonatal (e.g. hypoglycaemia and hypothermia [2–4]) and longer-term outcomes (e.g. hypertension, insulin resistance and obesity [5]).

An important consideration in the use of the reference charts is the population on which the charts were constructed. For example, ethnicity, period of data collection (secular changes), socioeconomic status and whether the sample adhered to a set of strict inclusion criteria (e.g. the WHO standard [6]) should all be considered. Several birth weight references have been produced in China [7, 8], however, limitations with the study design and/or the sample used have meant that no consensus has been reached regarding which reference should be adopted for clinical practice. Large socioeconomic disparities across different provinces within China make it inappropriate to use a common reference. These disparities lead to differences in morbidity and mortality in different areas. Accordingly, there is justification for the development of a set of birth weight references for different areas. Chongqing is a municipality in southwest China with a population in excess of 30 million [9] and annual births of approximate 350,000. A randomised controlled trial at The First Affiliated Hospital-Chongqing Medical University [10] was recently undertaken; the aims included the investigation of the effect of a nutritional intervention administered during gestation on a range of pregnancy outcomes (e.g. SGA, preterm birth and preeclampsia). When pregnancy outcomes were determined, a particularly low rate of SGA (≈ 2%) was identified, and it became apparent that the birth weight charts in routine clinical use were outdated and did not pertain to the Chongqing population. In light of this and given the specific demographic profile of Chongqing, we believe that a birth weight chart specific to Chongqing is required. Therefore, the aim of this study was to produce a new set of birth weight references, specific to Chongqing. We also sought to identify whether important differences exist in the birth weight distributions of those born in the central urban and non-urban districts and whether this necessitated the construction of separate charts.

## Methods

### Data source

Data were extracted from the Chongqing National Population-Based Birth Defects Surveillance System (CNPBDSS). The CNPBDSS is a subset of the National Population-Based Birth Defects Surveillance System (NPBDSS) which was established in 2006 by the Ministry of Health, China to record all livebirths, fetal deaths and stillbirths. The system covers 64 counties and districts under the central government. Further details have been described elsewhere [11, 12]. Gestational age was calculated using the date of the woman’s last menstrual period (LMP). If they could not remember the date of LMP, gestational age was calculated according to ultrasound examination at 12 gestational weeks. Birthweight was measured within 1 hour after birth by a professional midwife. Data were extracted for all singleton births delivered in 62 hospitals in the Chongqing municipality from 1st January, 2014 to 30th September, 2017. The hospitals included were obtained via cluster sampling at the administrative area level. Within the ‘Urban developed economic circle’, six hospitals were selected. For the other three administrative areas (‘Newly developed urban area’, ‘North-eastern ecological conservation area’ and the ‘South-eastern environment protection area’), two hospitals were selected from each district within the area, resulting in 22 hospitals from the ‘Newly developed urban area’, 22 from the ‘North-eastern ecological conservation area’ and 12 hospitals from the ‘South-eastern environment protection area’. For each year between 2014 and 2017, two hospitals were selected from each district, of which one was an urban hospital and the other was a rural hospital. The data from urban hospitals were collected through the whole course of data collection. As there are over 100 rural hospitals in each district, rural hospitals were re-selected randomly year by year, without resampling, to reduce selection bias.

### Patient and public involvement

We collected the data of 348,454 single births from 62 hospitals in Chongqing. Informed consent for use of the data was obtained when the Birth Defects Register Forms were collected [11, 12].

### Data analysis

#### Centile estimation

Analyses were restricted to live-born infants born between 28^+ 0^ and 42^+ 6^ weeks gestational age. Infants were not included if sex or birthweight was not recorded or undefined, or if the birthweight was considered as an outlier. An outlier birthweight was defined as being greater than four standard deviations from the median birthweight for their gestational age and sex.

An a priori decision was taken to use the LMS (Lambda, mu, sigma) method to calculate the new birth weight centiles [13], thus assuming birth weight has a Box-Cox Cole and Green distribution (BCCG).

The LMS method was implemented using the GAMLSS package in R version 3.2.3, as suggested by the World Health Organisation [14]. This approach converts the skewed distribution of birth weights to an appropriate Normal distribution by estimating and implementing the L (Box-Cox power), M (median) and S (coefficient of variation) parameters. In an effort to identify the optimum number of effective degrees of freedom (edf) for the penalised spline models needed to acquire the smoothed L, M and S curves (over gestational age), the automated *‘pb’* function was implemented, with gestational age on the raw scale (weeks). The *‘pb’* function automatically selects the edf which results in the best-fitting model, as determined, in this analysis, by the lowest value for the Bayesian Information Criterion (BIC). Accordingly, the resulted edf for the L, M and S parameters were 2.00, 8.52 and 5.93, respectively, for males and 3.57, 9.72 and 6.18 for females. Residual diagnostics (via checking de-trended Q-Q plot (‘worm plots’) parameters and the normality of residuals) were performed and suggested that the BCCG distribution provided a suitable fit to the data.

The smoothed values of L, M and S were then applied to transform the observed distribution of birthweight to an accepted Normal distribution. Using these LMS values, any birth weight centile could be outputted at any gestational age via the formula:

$$ y=M{\left[1+ LS{z}_{\alpha}\right]}^{\frac{1}{L}} $$

where *z*_*a*_ is the normal equivalent deviate corresponding to a given centile [13]. LMS values were exported from R and the final centile charts were plotted using StataIC version 14.

Post creating the centiles, we defined the percentage of births below the set of standard centile thresholds, both overall and by gestational age group: < 32 weeks (Very Preterm, VPT); 32–36 weeks (Late and Moderate Preterm, LMPT) and > 36 weeks (Term). For example, if the derived centiles calculate accurately, 10% of birthweights should fall below the 10th centile.

The decision to use the BCCG (LMS) distribution was motivated by a desire to be able to use the resulting charts to produce an exact centile or z-score for a given infant, which is not straightforward with other distributions (e.g. Box-Cox power exponential (BCPE) or Box-Cox T (BCT). However, as a sensitivity analysis, we utilised the ‘lms’ function in R which fits a number of distributions to the response variable (birth weight), in order to find the optimal distribution (lowest maximum likelihood). It was observed that the Box-Cox T distribution (BCT) provided the best fit to the data and we have reported the proportions of infants below the same set of centiles, as well as the weekly birth weights at these centiles, in the supplementary material.

In order to determine whether the distribution of birth weight was different in infants born to mothers who delivered in hospitals within the Urban developed economic circle (urban) compared to infants born to mothers who delivered outside of this area (i.e. in the Newly developed urban area, North-eastern ecological conservation area and the South-eastern environment protection area (rural), we repeated the above steps after incorporating a covariate for urbanicity, which resulted in the production of area-specific LMS parameters and birth weight centiles.

#### Comparison to Chinese ‘national reference’

We sought to compare the proposed centiles to those of the ‘national population-based references’ of Dai et al. (2014). Particularly, we calculated the absolute (gram) and percentage ((Dai et al. centile value - proposed centile value)/proposed centile value) *100) differences between the birth weight values at the 10th, 50th and 90th centiles of our charts to those of the Dai et al. charts. This was performed at 28–42 completed weeks of gestation because this was the period of overlap between the two charts.

## Results

A total of 339,239 singleton births occurred in the 62 hospitals sampled from the Chongqing municipality between 1st January, 2014 and 30th September, 2017. Of those, 785 singletons were excluded from analysis due to: an implausible birthweight (*n* = 452) or being born outside of 28–42 completed weeks (*n* = 333), leaving a total of 338,454 birth weights included in the analysis. The median birth weight of the sample was 3280 g (interquartile range (IQR): 3000; 3550), with a median gestation of 39 completed weeks (IQR: 38; 40).

### Creation of centiles

**Fig. 1:**
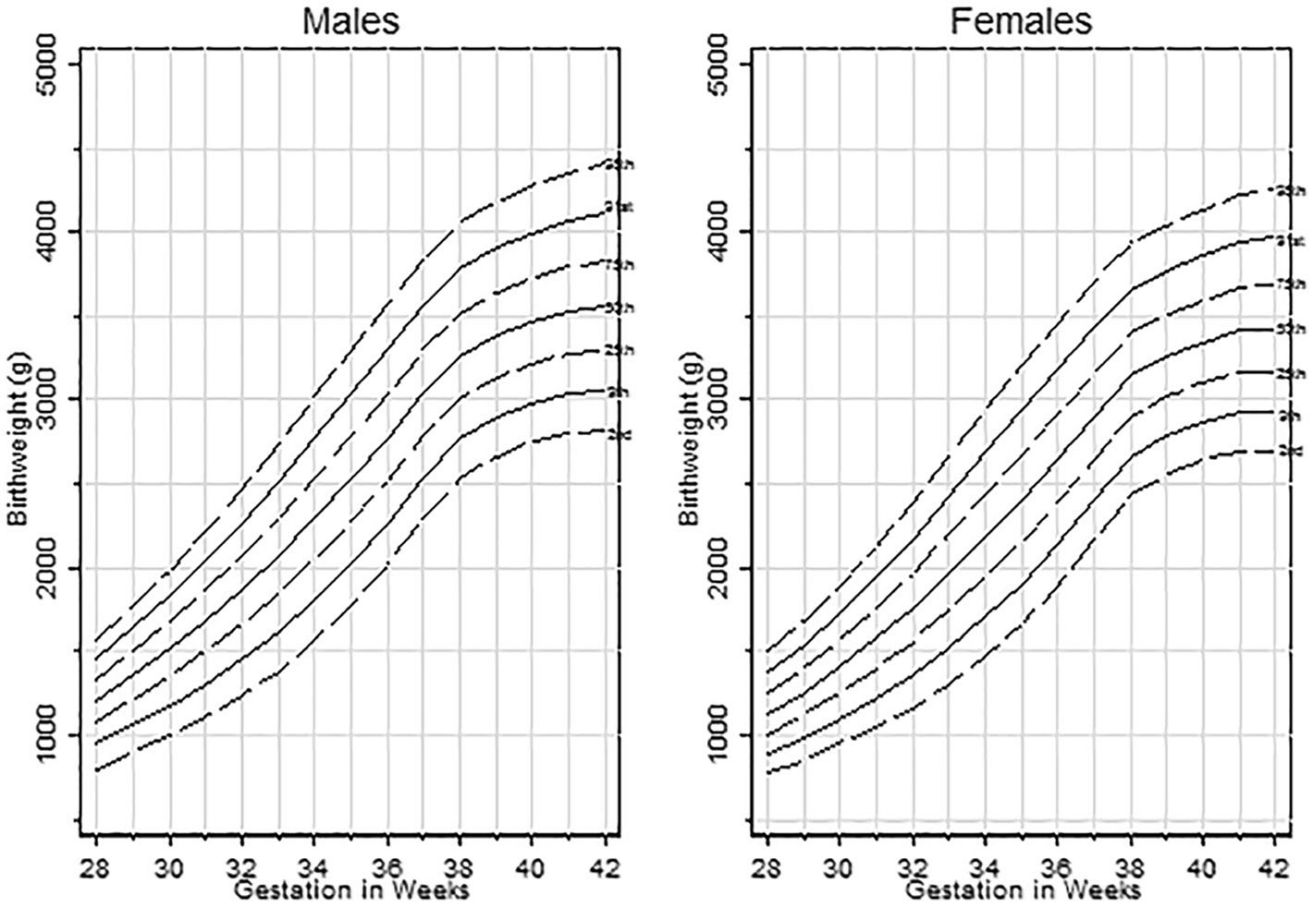
Birth weight centiles for males and females born in Chongqing (*n* = 338,454)

The LMS parameters were calculated (Additional file 1: Table S1) which allow estimation of any specified centile using the formula. Estimated centile charts are presented for males (Fig. 1-left panel) and females (Fig. 1-right panel) demonstrating the 0.4th, 2nd, 9th, 25th, 50th, 75th, 91st, 98th and 99.6th centiles. Table 1 shows the performance of the centiles in the 338,454 infants who were implemented to construct the charts, stratified by sex and gestational age. In both males and females, the new centiles performed adequately, with a similar portion of infants classified below each centile as to what would be expected. For example, looking at the 2nd centile, the new centiles classified 2.37% of males and 2.26% of females as below the 2nd centile (expected percentage = 2.28%). For the 98th centile, the new centiles classified 2.48% of males and 2.52% of females as above the 98th centile (expected percentage = 2.28%). The mew centiles generally performed better at term gestational ages. Additional file 1: Table S2 shows the proportions below the respective centiles when a BCT distribution was used for centile fitting. Whilst these centiles resulted in an observed proportion was closer to the expected proportion than that observed for the BCCG distribution, the differences were small.

**Table 1:** Expected and observed percentage (n) below selected centiles for validation of the birthweight mathematical model at very preterm, late-moderate preterm, term and all gestational ages

	Very preterm (< 32 weeks)			Late-moderate preterm (32–36 weeks)			Term (> 36 weeks)			All gestational ages (28–42 weeks)		
Expected^a^	Observed % (n): males (*n* = 852)	Observed % (n): females (*n* = 687)	Observed % (n): both sexes (*n* = 1539)	Observed % (n): males (*n* = 11,037)	Observed % (n): females (*n* = 9040)	Observed % (n): both sexes (*n* = 20,077)	Observed % (n): males (*n* = 164,513)	Observed % (n): females (*n* = 152,325)	Observed % (n): both sexes (*n* = 316,838)	Observed % (n): males (*n* = 176,402)	Observed % (n): females (*n* = 162,052)	Observed % (n): both sexes (*n* = 338,454)
Below 0.4th (0.38%)	0.35	0.58	0.45	0.72	0.73	0.73	0.52	0.56	0.54	0.54	0.57	0.55
Below 2nd (2.28%)	2.35	2.62	2.47	2.84	2.54	2.71	2.34	2.24	2.29	2.37	2.26	2.31
Below 9th (9.13%)	8.22	8.88	8.51	8.82	8.48	8.67	8.06	8.35	8.20	8.11	8.36	8.23
Below 25th (25.24%)	23.12	21.69	22.48	23.90	23.05	23.52	25.19	24.47	24.84	25.10	24.37	24.75
Below 50th (50%)	48.94	49.78	49.32	50.02	50.61	50.29	49.73	50.74	50.22	49.75	50.73	50.22
Below 75th (74.76%)	78.17	78.17	78.17	76.27	76.68	76.46	75.67	75.40	75.54	75.72	75.48	75.61
Below 91st (90.87%)	93.08	92.43	92.79	92.15	91.45	91.84	90.90	91.11	91.00	90.99	91.13	91.06
Below 98th (97.72%)	97.18	97.53	97.34	97.44	97.16	97.32	97.52	97.50	97.51	97.52	97.48	97.50
Below 99.6th (99.62%)	98.94	99.27	99.09	99.24	99.12	99.18	99.46	99.49	99.47	99.44	99.46	99.45

^a^Centiles and expected percentages based on 0.67 standard deviation score spacing between centile [15]

Weekly sex-specific birth weights at the 10th (representing small-for-gestational age (SGA)), 50th and 90th (representing large-for-gestational age (LGA)) centiles are presented in Additional file 1: Table S3. Additional file 1: Table S4 presents the sex-specific birth weights at the 10th, 50th and 90th centiles fitted using a BCT distribution. The differences between the distributions were small. For example, male birth weight at 28 weeks at the 10th, 50th and 90th centiles, assuming a BCCG distribution, were 959 g, 1214 g and 1445 g respectively, whereas assuming a BCT distribution they were 1002 g, 1221 g and 1430 g respectively. A similar sized difference in the estimated weights at these centiles was observed for females and across the range of gestational ages.

### Rural urban differences

**Fig. 2:**
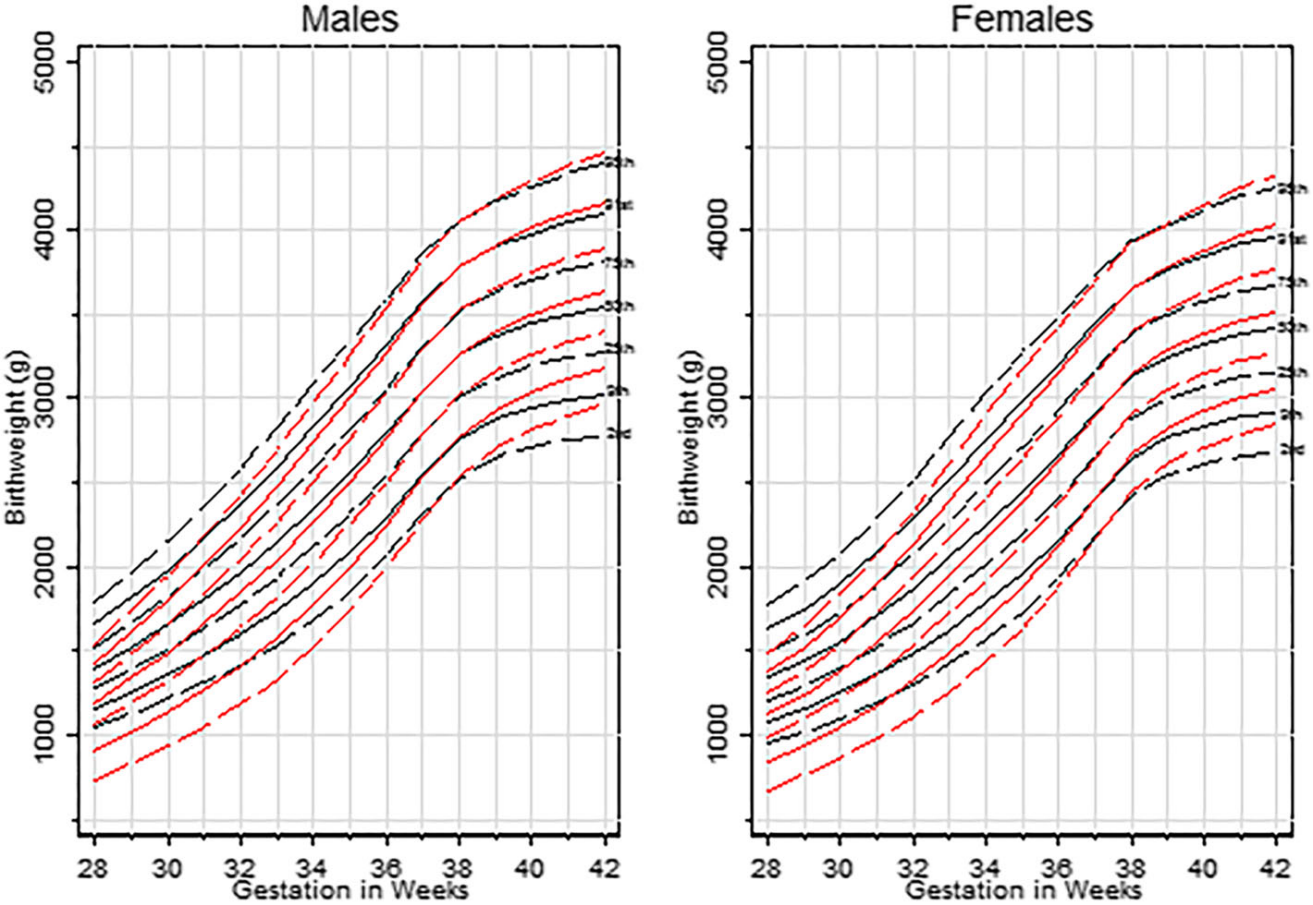
Birth weight centiles for infants born in ‘urban’ (red) vs rural (black) districts of Chongqing (left panel-males; right panel-females)

Urban births accounted for 39.47% (*n* = 133,596) of the total sample. Urban and rural sex-specific centiles are shown in Fig. 2. For both males and females, estimated centiles were lower for urban infants at preterm gestational ages (Table 2). For example, at the 28th week the 10th, 50th and 90th centiles for urban males represented birth weights of 919 g, 1191 g and 1421 g respectively, whilst the equivalent centiles for rural infants were 1172 g, 1402 g and 1650 g respectively. At term, the birth weight distributions shifted, such that centiles for urban infants were greater than the equivalent centiles for rural infants (Table 2). For example, at the 40th week the 10th, 50th and 90th centiles for urban males were 3051 g, 3502 g and 3994 g respectively, whereas the equivalent centiles for rural infants were 2968 g, 3447 g and 3958 g respectively.

**Table 2:** Weekly birth weights (g) at the 10th, 50th and 90th centiles: urban vs rural

	Males						Females					
Gestation (complete weeks)	Urban			Rural			Urban			Rural		
	Weight (g) at 10th centile	Weight (g) at 50th centile	Weight (g) at 90th centile	Weight (g) at 10th centile	Weight (g) at 50th centile	Weight (g) at 90th centile	Weight (g) at 10th centile	Weight (g) at 50th centile	Weight (g) at 90th centile	Weight (g) at 10th centile	Weight (g) at 50th centile	Weight (g) at 90th centile
28 + 0–28 + 6	919	1191	1421	1172	1402	1650	847	1120	1363	1089	1343	1619
29 + 0–29 + 6	1034	1341	1605	1271	1530	1808	943	1238	1506	1164	1438	1735
30 + 0–30 + 6	1157	1497	1796	1373	1663	1974	1060	1384	1684	1259	1560	1887
31 + 0–31 + 6	1288	1664	2002	1485	1809	2157	1193	1552	1891	1370	1705	2069
32 + 0–32 + 6	1436	1848	2226	1608	1971	2360	1341	1740	2123	1498	1869	2273
33 + 0–33 + 6	1605	2051	2470	1752	2152	2579	1511	1947	2375	1639	2052	2501
34 + 0–34 + 6	1799	2271	2726	1915	2349	2812	1699	2168	2638	1800	2250	2740
35 + 0–35 + 6	2019	2507	2989	2102	2563	3054	1904	2393	2891	1979	2451	2965
36 + 0–36 + 6	2270	2763	3261	2321	2797	3303	2137	2626	3133	2179	2660	3181
37 + 0–37 + 6	2549	3034	3536	2565	3045	3555	2420	2897	3399	2432	2908	3423
38 + 0–38 + 6	2792	3265	3763	2777	3254	3761	2691	3149	3636	2668	3136	3640
39 + 0–39 + 6	2952	3408	3896	2897	3375	3883	2843	3285	3761	2791	3248	3740
40 + 0–40 + 6	3051	3502	3994	2968	3447	3958	2940	3380	3859	2859	3321	3821
41 + 0–41 + 6	3132	3581	4078	3014	3504	4029	3022	3466	3956	2909	3383	3898
42 + 0–42 + 6	3193	3641	4147	3044	3542	4079	3075	3518	4014	2930	3411	3936

### Comparison to Chinese ‘national reference’

A consistent pattern was observed for both sexes when comparing the birth weights at the 10th and 50th centiles. This was characterised by higher birth weights at the 10th and 50th centiles of the proposed charts at 28 and 29 weeks, which lower birth weights between 30 and 37 weeks and finally, higher birth weights from 38 weeks onwards. A comparison of the respective 90th centiles revealed that the proposed centiles estimated lower birth weights from 28 to 37 weeks, with higher birth weights thereafter (Additional file 1: Table S5).

## Discussion

We have used routinely collected birth data to produce a contemporary set of birth weight centiles for Chongqing. Chongqing is a major municipality in China with an approximate population of 30 million and 350,000 births per year. These centiles, constructed using all singleton births occurring between 2014 and 2017, supply a contemporary and representative reference with which to more accurately identify newborns with suspected intrauterine growth restriction (IUGR). Newborns with suspected IUGR may benefit from carefully monitoring in the period subsequently following birth for conditions such as hypoglycaemia.

The centiles were produced using the LMS method [13]. Using these values, it is possible to generate the z-score and exact centile for any infant delivered between 28 and 42 completed weeks. Recently, ‘national population-based, gestational age-specific’ birth weight centiles have been produced in China [12], but the method employed does not allow for the subsequent generation of an individual’s z-score relative to the chart. Rather, weekly birth weights for a given centile were published and thus it was only possible to determine whether or not an infant was below a particular centile, without being able to quantify how far below the centile they are. The L, M and S parameters employed in our study greatly increase the application of the charts and provide a greater indication of the severity of any abnormal fetal growth.

We observed marked differences in the birth weight distribution of infants whose mothers delivered in ‘urban’ vs ‘rural’ areas. Specifically, in the preterm period (< 37 weeks) we observed markedly higher birth weights for a given centile in infants born in rural areas compared to urban areas. This pattern was reversed in term births, such that ‘urban’ infants had higher birth weights at equivalent centiles. The authors conclude that access to better antenatal care may explain this observation. We speculate that the main contributor to the differences between the two groups at the earlier gestational ages is due to selection bias created via the improved antenatal care experienced by those delivering in hospitals within the central Chongqing municipality. For example, an infant born at 28 weeks who is delivered in a hospital within the central Chongqing municipality with access to more advanced healthcare facilities, has an increased likelihood of survival than the same baby born in a rural area. As centiles are based on live births, this would permit this lower birth weight to contribute to the urban centiles, whereas only the heavier (and thus more likely to survive) rural babies contribute to the rural centiles, thus shifting the birth weight distribution upwards at these earlier gestational ages. We hypothesise that some of the urban-rural variation could also be the result of differences in the proportion of pregnancies dated via ultrasonography. It has been shown that birth weights derived from ultrasound dated pregnancies have a higher population mean and their curves show less flattening at term than birth weights from pregnancies dated using LMP [16]. In addition to differences in antenatal care received between urban vs. rural women, a reduced income and education in rural women may also result in malnutrition, particularly anaemia [17], which may lead to intrapartum complications and thus an increased perinatal mortality rate in this group.

Cost is considered the main obstacle to the uptake of maternal care, particularly for poor rural households. Since the economic reforms introduced in the 1980s, which saw central government decentralise fiscal responsibility to local government and a concomitant decline in central government transfers into the health sector [18–21], health insurance coverage has decreased. The government has since responded to these concerns and in 1995 passed a law (Law on Maternal and Infant Health Care) which guarantees a woman’s right to deliver in hospital. Further programs, e.g. the National Programme to Reduce Maternal Mortality and Eliminate Neonatal Tetanus and the New Cooperative Medical Scheme (NCMS) have also been implemented since the year 2000, with the former aiming to improve infrastructure and staff training in lower-level hospitals and provide subsidies to encourage hospital deliveries, whilst the latter provides further reimbursement for maternity care. The evidence regarding whether these initiatives have reduced the inequalities in access to maternal and obstetric care is inconsistent [22, 23].

At term, the direction of the urban – rural birth weight difference was reversed. We speculate that this is likely the result of better nutritional status of women in urban areas which may have resulted in increased birth weight.

### Limitations

Due to the low number of births in our cohort that occurred in the earlier gestational weeks (24–27 weeks; *n* = 112), we did not have enough data to estimate reliable centiles at these gestational weeks. As such, in those periods where there were less data, the performance of the centiles was poor. Despite the charts constructed assuming a BCT distribution (which additionally accounts for kurtosis in the distribution) resulting in improved performance at the earlier weeks, the differences were generally small. Besides, antenatal characteristics of the mother are not known.

We classified women as either ‘urban’ or ‘rural’ based on whether they delivered in the Urban developed economic circle, as we did not have information on home address. Whilst this provides an indication of the likely level of antenatal care received, we were unable to determine whether women were residing in the same areas that they delivered. It was therefore not possible to determine the possible socioeconomic and environmental exposures experienced by the women, which may have led to the observed differences in the birth weight distributions.

## Conclusions

We have constructed a readily utilizable set of birth weight references from a large representative sample of births in Chongqing. Unlike former charts, we have utilised a method which allows for calculation of the exact birth weight centile for any infant delivered between 28 and 42 completed weeks. Specifically, the L, M and S parameters published here can be easily employed (using the formula provided) to provide a greater indication of the severity of abnormal fetal growth.

## Supplementary information

**Additional file 1: Table S1.** L, M and S values to produce any specified centile by sex and completed week of gestation. **Table S2.** Expected and observed percentage (n) below selected centiles: very preterm, late-moderate preterm, term, all gestational ages (BCT distribution). **Table S3.** Weekly birth weights (g) at the 10th, 50th and 90th centile. **Table S4.** Weekly birth weights (g) at the 10th, 50th and 90th centile (BCT distribution). **Table S5.** Comparison of new centiles vs Dai et al. (2014)a centiles.

## Data Availability

The datasets generated and/or analyzed during the current study are available from the corresponding author upon reasonable request.

## Abbreviations

BCCG distribution: Box-Cox Cole and Green distribution
BCPE: Box-Cox power exponential
BCT: Box-Cox T
BIC: Bayesian Information Criterion
CNPBDSS: Chongqing National Population-Based Birth Defects Surveillance System
GDP: Gross-Domestic Product
IQR: interquartile range
IUGR: intrauterine growth restriction
LGA: large-for-gestational age
LMP: last menstrual period
NCMS: New Cooperative Medical Scheme
NMR: neonatal mortality rates
NPBDSS: The National Population-Based Birth Defects Surveillance System
SGA: small-for-gestational age
