# Supplementary material for: Birth weight charts for a Chinese population: an observational study of routine newborn weight data from Chongqing
Source: BMC Pediatr. 2019 Nov 11;19:426. doi: 10.1186/s12887-019-1816-9 (PMC6844044; doi:10.1186/s12887-019-1816-9)
Supplement: Supplementary file 1 — Additional file 1: Table S1. L, M and S values to produce any specified centile by sex and completed week of gestation. Table S2. Expected and observed percentage (n) below selected centiles: very preterm, late-moderate preterm, term, all gestational ages (BCT distribution). Table S3. Weekly birth weights (g) at the 10th, 50th and 90th centile. Table S4. Weekly birth weights (g) at the 10th, 50th and 90th centile (BCT distribution). Table S5. Comparison of new centiles vs Dai et al. (2014)a centiles. [file 12887_2019_1816_MOESM1_ESM.pdf]

Supplementary table 1: L, M and S values to produce any specified centile by sex and completed week of gestation

Gestation	**Males**				**Females**			
	n	***L***	***M***	***S***	*n*	***L***	***M***	***S***
28+0- 28+6	115	1.495	1214	0.155	74	0.768	1129	0.164
29+0- 29+6	155	1.413	1365	0.158	116	0.812	1257	0.166
30+0- 30+6	219	1.330	1520	0.161	192	0.85	1409	0.168
31+0- 31+6	363	1.247	1686	0.163	305	0.878	1578	0.171
32+0- 32+6	562	1.164	1869	0.164	451	0.892	1764	0.173
33+0- 33+6	931	1.081	2073	0.163	713	0.889	1969	0.173
34+0- 34+6	1533	0.998	2292	0.158	1313	0.869	2190	0.169
35+0- 35+6	2595	0.915	2526	0.150	2169	0.827	2414	0.161
36+0-36+6	5416	0.832	2777	0.140	4394	0.763	2642	0.148
37+0- 37+6	14869	0.749	3040	0.127	11750	0.676	2904	0.133
38+0- 38+6	38056	0.666	3260	0.117	31229	0.577	3142	0.119
39+0- 39+6	58593	0.583	3388	0.112	54863	0.495	3262	0.113
40+0- 40+6	39803	0.500	3467	0.11	39953	0.452	3342	0.111
41+0- 41+6	12333	0.417	3528	0.11	13614	0.442	3411	0.112
42+0- 42+6	859	0.334	3555	0.112	916	0.444	3425	0.114

Supplementary table 2: Expected and observed percentage (n) below selected centiles: very preterm, late-moderate preterm, term, all gestational ages (BCT distribution)

	Very preterm (<32 weeks)			*Late-moderate preterm (32-36 weeks)*			*Term (>36 weeks)*			*All gestational ages (28-42 weeks)*		
Expected†	Observed % (n): males (n=852)	Observed % (n): females (n=687)	Observed % (n): both sexes (n=1,539)	Observed % (n): males (n=11,037)	Observed % (n): females (n=9,040)	Observed % (n): both sexes (n=20,077)	Observed % (n): males (n=164,513)	Observed % (n): females (n=152,325)	Observed % (n): both sexes (n=316,838)	Observed % (n): males (n=176,402)	Observed % (n): females (n=162,052)	Observed % (n): both sexes (n=338,454)
Below 0.4th (0.38%)	0.12 (1)	0.15 (1)	0.13 (2)	0.40 (44)	0.40 (36)	0.40 (80)	0.44 (722)	0.45 (681)	0.44 (1,403)	0.43 (767)	0.44 (718)	0.44 (1,485)
Below 2nd (2.28%)	1.53 (13)	1.89 (13)	1.69 (26)	2.17 (239)	1.88 (170)	2.04 (409)	2.06 (3,396)	2.00 (3,041)	2.03 (6,437)	2.07 (3,648)	1.99 (3,224)	2.03 (6,872)
Below 10th (10.0%)	11.15 (95)	11.79 (81)	11.41 (176)	9.65 (1,065)	10.02 (906)	9.82 (1,971)	10.08 (16,591)	9.56 (14,558)	9.83 (31,149)	10.06 (17,751)	9.59 (15,545)	9.84 (33,296)
Below 25th (25.24%)	25.23 (215)	24.75 (170)	25.02 (385)	24.66 (2,722)	23.86 (2,157)	24.30 (4,879)	25.40 (41,788)	26.37 (40,165)	25.87 (81,953)	25.35 (44,725)	26.22 (42,492)	25.77 (87,217)
Below 50th (50%)	49.06 (418)	49.93 (343)	49.45 (761)	50.01 (5,520)	50.37 (4,553)	50.17 (10,073)	49.85 (82,012)	50.25 (76,537)	50.04 (158,549)	49.86 (87,950)	50.25 (81,433)	50.05 (169,383)
Below 75th (74.76%)	74.30 (633)	76.13 (523)	75.11 (1,156)	75.75 (8,360)	74.51 (6,736)	75.19 (15,096)	75.20 (123,718)	75.07 (114,347)	75.14 (238,065)	75.23 (132,711)	75.04 (121,606)	75.14 (25,4317)
Below 90th (90.0%)	91.67 (781)	90.10 (619)	90.97 (1,400)	90.25 (9,961)	89.56 (8,096)	89.94 (18,057)	89.78 (147,703)	89.86 (136,872)	89.82 (284,575)	89.82 (158,445)	89.84 (145,587)	89.83 (304,032)
Below 98th (97.72%)	97.89 (834)	98.11 (674)	97.99 (1,508)	98.01 (10,817)	97.81 (8,842)	97.92 (19,659)	97.97 (161,168)	97.87 (149,084)	97.92 (310,252)	97.97 (172,819)	97.87 (158,600)	97.92 (331,419)
Below 99.6th (99.62%)	99.65 (849)	99.42 (683)	99.55 (1,532)	99.52 (10,984)	99.56 (9,000)	99.54 (19,984)	99.62 (163,892)	99.61 (151,731)	99.62 (315,623)	99.62 (175,725)	99.61 (161,414)	99.61 (337,139)

Supplementary table 3: Weekly birth weights (g) at the 10th, 50th and 90th centile

Gestation	**Males**			**Females**		
	Weight (g) at 10th centile	***Weight (g) at 50th centile***	***Weight (g) at 90th centile***	***Weight (g) at 10th centile***	***Weight (g) at 50th centile***	***Weight (g) at 90th centile***
28+0- 28+6	959	1214	1445	898	1129	1372
29+0- 29+6	1075	1365	1631	995	1257	1530
30+0- 30+6	1194	1520	1824	1111	1409	1717
31+0- 31+6	1324	1686	2030	1237	1578	1928
32+0- 32+6	1469	1869	2256	1378	1764	2160
33+0- 33+6	1636	2073	2503	1538	1969	2411
34+0- 34+6	1828	2292	2756	1723	2190	2671
35+0- 35+6	2045	2526	3015	1925	2414	2921
36+0-36+6	2287	2777	3282	2153	2642	3154
37+0- 37+6	2556	3040	3545	2423	2904	3412
38+0- 38+6	2784	3260	3761	2678	3142	3637
39+0- 39+6	2916	3388	3889	2807	3262	3752
40+0- 40+6	2995	3467	3973	2885	3342	3836
41+0- 41+6	3051	3528	4046	2941	3411	3920
42+0- 42+6	3069	3555	4090	2945	3425	3946

Supplementary table 4: Weekly birth weights (g) at the 10th, 50th and 90th centile (BCT distribution)

Gestation	Males			Females		
	Weight (g) at 10th centile	*Weight (g) at 50th centile*	*Weight (g) at 90th centile*	*Weight (g) at 10th centile*	*Weight (g) at 50th centile*	*Weight (g) at 90th centile*
28+0- 28+6	1002	1221	1430	907	1131	1374
29+0- 29+6	1097	1361	1611	1007	1237	1474
30+0- 30+6	1215	1517	1803	1161	1431	1704
31+0- 31+6	1348	1685	2005	1268	1581	1897
32+0- 32+6	1489	1868	2228	1400	1762	2131
33+0- 33+6	1655	2076	2481	1555	1961	2380
34+0- 34+6	1850	2298	2737	1751	2191	2648
35+0- 35+6	2062	2529	2998	1954	2416	2898
36+0-36+6	2293	2771	3263	2162	2639	3139
37+0- 37+6	2561	3036	3534	2428	2898	3395
38+0- 38+6	2799	3262	3754	2696	3144	3627
39+0- 39+6	2924	3385	3879	2816	3259	3740
40+0- 40+6	3000	3466	3967	2890	3341	3832
41+0- 41+6	3062	3534	4042	2948	3414	3915
42+0- 42+6	3030	3529	4068	2927	3412	3928

Supplementary table 5 Comparison of new centiles vs Dai et al (2014)[a] centiles

GA at birth (weeks)	Birth weight centile (g)																	
	Males									Females								
	10th [b]	Dai et al 10th	Diff (g, (%)[c])	50th [b]	Dai et al 50th	Diff (g, (%)[c])	90th [b]	Dai et al 90th	Diff (g, (%)[c])	10th [b]	Dai et al 10th	Diff (g, (%)[c])	50th [b]	Dai et al 50th	Diff (g, (%)[c])	90th [b]	Dai et al 90th	Diff (g, (%)[c])
28^{+0-6}	959	895	-64 (-6.7)	1214	1152	-62 (-5.1)	1445	1458	13 (0.9)	898	830	-68 (-7.6)	1129	1102	-27 (-2.4)	1372	1432	60 (4.4)
29^{+0-6}	1075	1056	-19 (-1.8)	1365	1355	-10 (-0.7)	1631	1707	76 (4.7)	995	992	-3 (-0.3)	1257	1309	52 (4.1)	1530	1692	162 (10.6)
30^{+0-6}	1194	1220	26 (2.2)	1520	1558	38 (2.5)	1824	1957	133 (7.3)	1111	1157	46 (4.1)	1409	1517	108 (7.7)	1717	1950	233 (13.6)
31^{+0-6}	1324	1388	64 (4.8)	1686	1765	79 (4.7)	2030	2208	178 (8.8)	1237	1325	88 (7.1)	1578	1726	148 (9.4)	1928	2206	278 (14.4)
32^{+0-6}	1469	1563	94 (6.4)	1869	1977	108 (5.8)	2256	2462	206 (9.1)	1378	1499	121 (8.8)	1764	1937	173 (9.8)	2160	2459	299 (13.8)
33^{+0-6}	1636	1746	110 (6.7)	2073	2194	121 (5.8)	2503	2717	214 (8.5)	1538	1680	142 (9.2)	1969	2150	181 (9.2)	2411	2707	296 (12.3)
34^{+0-6}	1828	1939	111 (6.1)	2292	2417	125 (5.5)	2756	2972	216 (7.8)	1723	1871	148 (8.6)	2190	2365	175 (8.0)	2671	2945	274 (10.3)
35^{+0-6}	2045	2143	98 (4.8)	2526	2642	116 (4.6)	3015	3215	200 (6.6)	1925	2071	146 (7.6)	2414	2578	164 (6.8)	2921	3167	246 (8.4)
36^{+0-6}	2287	2356	69 (3.0)	2777	2860	83 (3.0)	3282	3434	152 (4.6)	2153	2279	126 (5.9)	2642	2783	141 (5.3)	3154	3360	206 (6.5)
37^{+0-6}	2556	2565	9 (0.4)	3040	3058	18 (0.6)	3545	3613	68 (1.9)	2423	2482	59 (2.4)	2904	2967	63 (2.2)	3412	3515	103 (3.0)
38^{+0-6}	2784	2739	-45 (-1.6)	3260	3215	-45 (-1.4)	3761	3746	-15 (-0.4)	2678	2652	-26 (1.0)	3142	3115	-27 (-0.9)	3637	3632	-5 (-0.1)
39^{+0-6}	2916	2849	-67 (-2.3)	3388	3317	-71 (-2.1)	3889	3836	-53 (-1.4)	2807	2764	-43 (-1.5)	3262	3216	46 (-1.4)	3752	3717	-35 (-0.9)
40^{+0-6}	2995	2908	-87 (-2.9)	3467	3382	-85 (-2.5)	3973	3907	-66 (-1.7)	2885	2824	-61 (-2.1)	3342	3281	-61 (-1.8)	3836	3787	-49 (-1.3)
41^{+0-6}	3051	2943	-108(-3.5)	3528	3437	-91 (-2.6)	4046	3987	-59 (-1.5)	2941	2854	-87 (-3.0)	3411	3332	-79 (-2.3)	3920	3863	-57 (-1.5)
42^{+0-6}	3069	2963	-106(-3.5)	3555	3483	-72 (-2.0)	4090	4064	-26 (-0.6)	2945	2868	-77 (-2.6)	3425	3371	-54 (-1.6)	3946	3933	-13 (-0.3)

[a] from Dai et al, 2014;*Plos One*, Aug;9(8).

[b] Completed weeks e.g. values for 38 weeks are those born ≥38 and <39 weeks

[c] % difference= ((Dai et al centile value - proposed centile value)/proposed centile value) *100
